# Epigenetic Regulation of Glucose Transporters in Non-Small Cell Lung Cancer

**DOI:** 10.3390/cancers3021550

**Published:** 2011-03-25

**Authors:** Kenneth J. O'Byrne, Anne-Marie Baird, Lisa Kilmartin, Jennifer Leonard, Calen Sacevich, Steven G. Gray

**Affiliations:** Department of Clinical Medicine, Thoracic Oncology Research Group, Institute of Molecular Medicine, Trinity Centre for Health Sciences, St James's Hospital, Dublin 8, Ireland; E-Mails: kobyrne@stjames.ie (K.J.O′B.); bairda@tcd.ie (A.-M.B.) leonarje@tcd.ie (J.L.); Calen.Sacevich@nosm.ca (C.S.), lisa.kilmartin@ucd.ie (L.K.)

**Keywords:** NSCLC, Glucose transporter, epigenetics

## Abstract

Due to their inherently hypoxic environment, cancer cells often resort to glycolysis, or the anaerobic breakdown of glucose to form ATP to provide for their energy needs, known as the Warburg effect. At the same time, overexpression of the insulin receptor in non-small cell lung cancer (NSCLC) is associated with an increased risk of metastasis and decreased survival. The uptake of glucose into cells is carried out via glucose transporters or GLUTs. Of these, GLUT-4 is essential for insulin-stimulated glucose uptake. Following treatment with the epigenetic targeting agents histone deacetylase inhibitors (HDACi), GLUT-3 and GLUT-4 expression were found to be induced in NSCLC cell lines, with minimal responses in transformed normal human bronchial epithelial cells (HBECs). Similar results for GLUT-4 were observed in cells derived from liver, muscle, kidney and pre-adipocytes. Bioinformatic analysis of the promoter for GLUT-4 indicates that it may also be regulated by several chromatin binding factors or complexes including CTCF, SP1 and SMYD3. Chromatin immunoprecipitation studies demonstrate that the promoter for GLUT-4 is dynamically remodeled in response to HDACi. Overall, these results may have value within the clinical setting as (a) it may be possible to use this to enhance fluorodeoxyglucose (18F) positron emission tomography (FDG-PET) imaging sensitivity; (b) it may be possible to target NSCLC through the use of HDACi and insulin mediated uptake of the metabolic targeting drugs such as 2-deoxyglucose (2-DG); or (c) enhance or sensitize NSCLC to chemotherapy.

## 1. Introduction

Tumor bioenergetics is coming more and more into focus as data accumulates linking cancer genes, bioenergetics and malignant transformation. For most of their energy needs, normal cells rely on a process called respiration, which consumes oxygen and glucose to make energy-storing molecules of adenosine triphosphate (ATP). Cancer cells, however, typically resort to a different mechanism, glycolysis: the anaerobic breakdown of glucose into ATP. This increased glycolysis, (even in the presence of available oxygen), is known as the Warburg effect [1]. The rate of glycolysis in tumor cells can be more than 30-times greater than that of normal cells [2], and it is estimated that approximately 63% of the energy requirements of a malignant cell is produced in this way [3].

Glucose transporters (GLUTs) facilitate the uptake of glucose across the phospholipid membrane and into the cell along a concentration gradient. To date there have been 14 members of the GLUT family characterized and these are structurally divided into three classes. Class 1 includes GLUT 1-4, class 2 is comprised of GLUT 5, 7, 9, and 11, and class 3 consists of GLUT 6, 8, 10, and 12. All three classes share a high level of homology and a similar structure [4,5]. GLUT1 is responsible for the majority of basal glucose uptake and is a chief glucose transporter of viable non-small cell lung cancer (NSCLC) cells [6]. GLUT1 and GLUT-3 have been shown to be over-expressed in lung cancers among others [7]. GLUT-4 is an insulin dependent glucose transporter first described in rat skeletal muscle by Birnbaum [8]. It is generally only expressed in muscle and adipose tissue, and is typically stored in intracellular lipid rafts in these cells, and rapidly translocates to the plasma membrane in response to insulin signaling [9]. GLUT-4 is not typically expressed in normal lung cells [10], but can be observed in the developing lung [11]. It has however, also been reported that in approximately 6% of lung carcinoma samples GLUT-4 expression can be observed in regenerating alveolar and bronchiolar epithelia around and in the cancer tissues [12].

A potential link between the insulin signaling pathway and lung cancer came from a study showing that overexpression of the insulin receptor in lung cancer is associated with increased risk of metastasis and decreased survival [13]. This has led to the hypothesis that inhaled insulin may have potential for use in the early detection of lung cancer [14].

Aberrant epigenetic regulation is a frequent event in NSCLC and both altered DNA CpG methylation and histone post-translational modifications (PTMs) have been shown to have both predictive and prognostic significance in this disease [15]. There is some evidence that Glut-4 may be epigenetically downregulated via histone PTMs at its promoter in the muscles of intrauterine growth-restricted (IUGR) mice [16].

We examined the expression of Glut-4 in a small series of primary NSCLC tumor samples and an additional panel of NSCLC cell lines, and whether chromatin remodeling plays a role in the regulation of Glut-4 expression in this disease. We confirm that Glut-4 mRNA is upregulated in a small subset of NSCLC tumors. Our results also clearly indicate that Glut 3 and Glut-4 are regulated via histone PTMs in NSCLC. Furthermore, we also show that the zinc-finger factor CCCTC-Binding Factor (CTCF) may be a regulator of GLUT-4 expression. Our data indicate that GLUT-4 may be a suitable candidate target for both epigenetic therapy and/or metabolic targeting in the management and treatment of NSCLC.

## 2. Results and Discussion

### 2.1. Expression of Glut-3 and Glut-4 in Primary Lung Cancer Tumor Specimens and NSCLC Cell Lines

We examined the expression of Glut-4 in a panel of primary NSCLC tumor surgical specimens, comparing expression in the tumor *versus* matched normal lung tissue obtained from the same individuals. In agreement with previously published immunohistochemistry results [12], Glut-4 mRNA was detected in only two of the NSCLC samples or approximately 10% of samples (data not shown). In contrast, Glut-3 mRNA was detectable in all NSCLC samples, but no significant differences in mRNA levels were observed (data not shown).

### 2.2. Histone Acetylation is Involved in Regulating Glut-4

**Figure 1.**
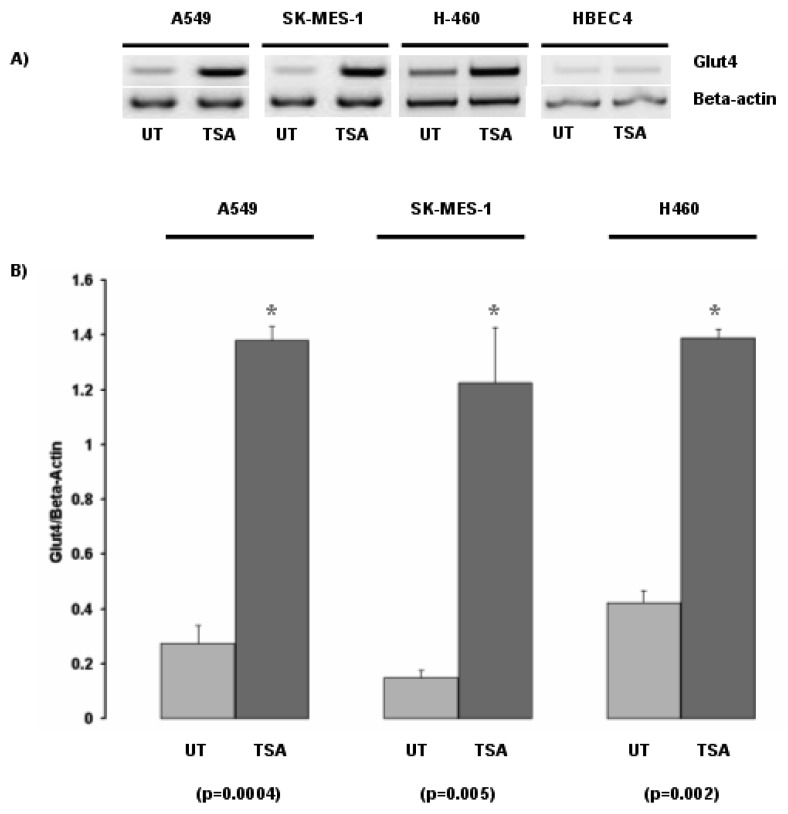

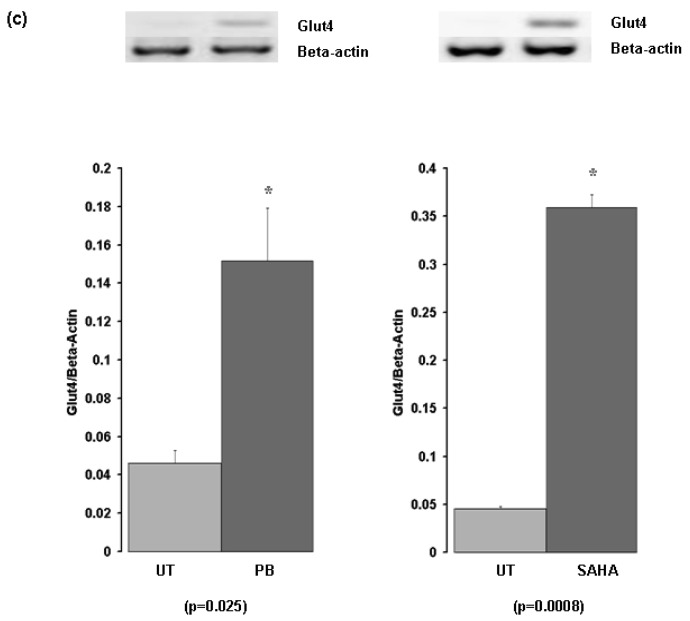
The histone deacetylase inhibitor trichostatin A induces Glut-4 in non-small cell lung cancer (NSCLC) cell lines. **(a)** The NSCLC cell lines A549, SK-MES-1, H-460 and the transformed normal bronchial epithelial cell line HBEC4 were treated with or without the histone deacetylase inhibitor Trichostatin A (TSA), and expression of Glut-4 mRNA examined by RT-PCR. **(b)** Densitometric analysis of Glut-4 expression. Beta-actin levels were used for normalization purposes. Data is expressed as mean ± SEM. Statistical analysis was performed using a Student's t test. **(c)** The A549 cell line was treated with or without the histone deacetylase inhibitors PB (Phenylbutyrate) or Vorinostat (SAHA), and expression of Glut-4 mRNA examined by RT-PCR. Data is expressed as mean ± SEM. Statistical analysis was performed using a Student's t-test. (UT, untreated; TSA, Trichostatin A; PB, phenylbutyrate; SAHA, Vorinostat).

Inhibition of histone deacetylases by the histone deacetylase inhibitor Trichostatin A (TSA) led to the induction of Glut-4 in the NSCLC cell lines A549, SK-MES-1 and H460 (Figure 1). In contrast, no major change in Glut-4 expression was observed in the normal bronchial epithelial cell line HBEC4 when exposed to TSA.

Subsequently we examined the response of Glut-4 to TSA with two other histone deacetylase inhibitors, Phenylbutyrate (PB), and the FDA approved HDAC inhibitor suberoylanilide hydroxamic acid (SAHA –Vorinostat) (Figure 1). We confirmed the initial observation that Glut-4 expression can be induced or increased following inhibition of histone deacetylases (Figure 1).

### 2.3. Histone Acetylation is also Involved in Regulating Glut-3

**Figure 2.**
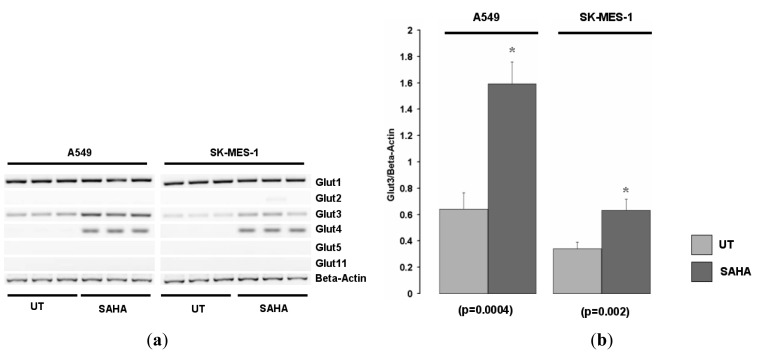
The histone deacetylase inhibitor Vorinostat (SAHA) induces only Glut 3 and Glut-4 in NSCLC cell lines. (**a**) A549 and SK-MES-1 cell lines were treated with or without the histone deacetylase inhibitor Vorinostat (SAHA), and mRNA expression of various Glut family members were examined by RT-PCR. (**b**) Densitometric analysis of Glut-3 expression. Beta-actin levels were used for normalization purposes. Data is expressed as mean ± SEM. Statistical analysis was performed using a Student's t test. (UT, untreated; SAHA, Vorinostat).

We examined the expression of various other Glut family members in the A549 and SK-MES-1cell lines for responses to HDACi. Glut1 was expressed in both cell lines but was not induced by SAHA (Figure 2a, and data not shown). Of the other Glut family members screened, Glut-3 was also found to be increased in these cell lines in response to HDAC inhibition (Figure 2b).

### 2.4. The Induction of Glut-4 in Other Tissues

**Figure 3.**
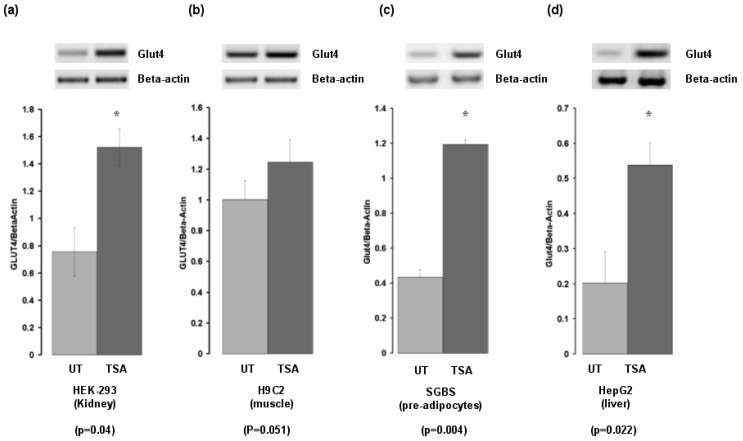
Effects of histone deacetylase inhibitors (HDACi) on Glut-4 in other cell lines. Various cell lines (**a**) HEK-293 (Kidney) (**b**) H9C2 (muscle), (**c**) SGBS (pre-adipocyte) and (**d**) HepG2 (liver) were treated with or without the histone deacetylase inhibitor Trichostatin A (TSA), and mRNA expression of Glut-4 was examined by RT-PCR. Densitometric analysis of Glut-4 expression with Beta-actin levels used for normalization purposes was carried out for each. Data is expressed as mean ± SEM. Statistical analysis was performed using a Student's t test. (UT, untreated; TSA, Trichostatin A).

As both tissue and inhibitor specific responses have been demonstrated to occur for HDACi [17], we examined the effect of TSA on Glut-4 expression in cell lines from organs important for insulin-mediated responses. Glut-4 was induced in cells derived from liver, pre-adipocytes, kidney, but was not induced in cells derived from skeletal muscle (Figure 3).

### 2.4. Regulation of Glut-4 Occurs through Direct Chromatin Remodeling

**Figure 4.**
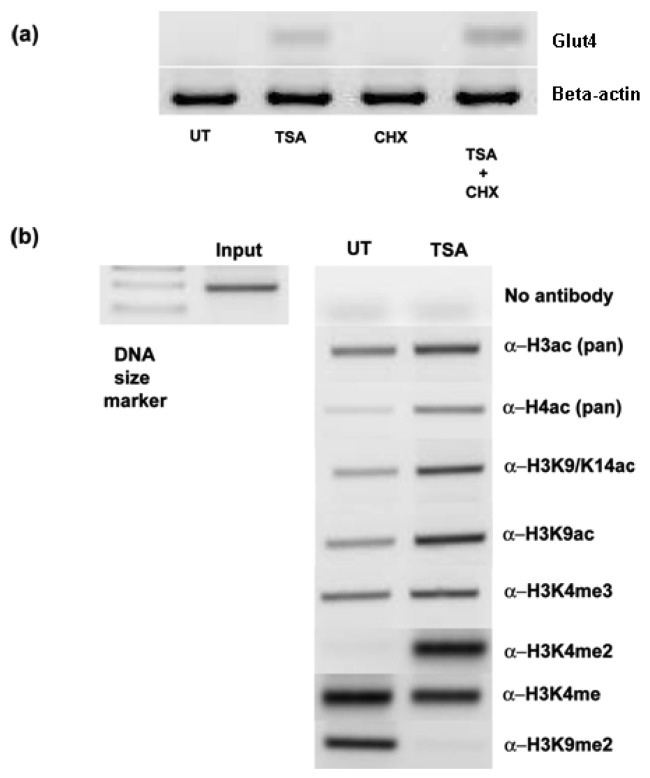
Direct chromatin remodeling of the Glut-4 promoter. (**a**) Pre-treatment with cycloheximide is unable to prevent TSA mediated induction of Glut-4 indicating that *de novo* protein synthesis is not required for activation that induction is via direct chromatin remodeling. (**b**) A chromatin immunoprecipitation (ChIP) assay demonstrates that TSA treatment results in an increase in the acetylation of histone H3 and H4 at the Glut-4 promoter. A549 cells were cultured in the presence or absence of TSA (250 ng/mL) for a period of 16 h. Subsequently a ChIP assay was performed using the following antibodies; pan acetylated histone H3 (Ac H3) and H4 (AcH4), histone H3 acetylated at lysine 9 and 14 (H3K9/K14Ac), histone H3 acetylated at lysine 9 (H3K9Ac), histone H3 tri-methylated at lysine 4 (H3K4me3), histone H3 di-methylated at lysine 4 (H3K4me2), histone H3 mono-methylated at lysine 4 (H3K4me3), histone H3 di-methylated at lysine 9 (H3K9me2). Input represents a positive control consisting of 1/10th of the original fixed chromatin prior to immunoprecipitation as recommended by the manufacturer (Diagenode). A no-antibody control was included to test for non-specific carriage of DNA with histones. (UT, untreated; CHX, cycloheximide; TSA, Trichostatin A).

To see if the induction/upregulation of Glut-4 was a direct consequence of HDAC inhibition, cells were exposed to cycloheximide prior to TSA treatments. Pretreatment with cycloheximide did not prevent the induction of Glut-4 mRNA (Figure 4a), suggesting that *de novo* protein synthesis was not required for the upregulation of Glut-4, and that direct chromatin remodeling of the Glut-4 promoter was occurring. To confirm that the observed effects of HDAC inhibition were due to increased histone hyperacetylation at the promoter of Glut-4, we carried out chromatin immunoprecipitation (ChIP) analysis at the Glut-4 promoter in A549 cells treated with TSA. A no-antibody control was included to test for non-specific carriage of DNA with histones. As can be seen in Figure 4b, treatment with TSA results in an increase in the amount of PCR product for Glut-4 indicating an increase in histone hyperacetylation around its promoter. Using specific antibodies we show that lysine 9 and lysine 14 are hyperacetylated in this region following treatment with TSA, and clearly demonstrate that chromatin remodeling is directly involved with the activation of Glut-4. Interestingly, no enhancement of histone trimethylation on lysine 4 of histone H3 was observed in response to promoter activation. H3K4me3 is considered to be a mark for transcriptionally active chromatin [18], and recent findings suggest that methylated H3K4 acts to facilitate the competency of pre-mRNA maturation through the bridging of spliceosomal components to H3K4me3 via CHD1 [19]. Unusually, we observe a strong increase in the levels of histone H3 lysine 4 dimethylation (H3K4me2) a modification generally perceived to be associated with repressive chromatin [20], at the Glut-4 promoter following activation of transcription via HDACi (Figure 4). However, this mark has also been found to be enriched in the *cis*-regulatory regions of active promoters as well as at developmentally poised genes [21]. We also observe a loss of an activating mark for transcription, histone lysine 4 monomethylation (H3K4me) [22], following activation of Glut-4 in our cells. In agreement with the current literature, we observe the presence of a repressive mark H3K9Me2 at the Glut-4 promoter prior to activation via HDACi, and levels of this histone modification are lost following activation [20,22]. This would therefore indicate that much more detailed studies of the histone post-translational modifications will be required to functionally elucidate the “histone code” regulating this promoter region.

### 2.5. Effect of Glut-4 Induction by HDACi Over Time

**Figure 5.**
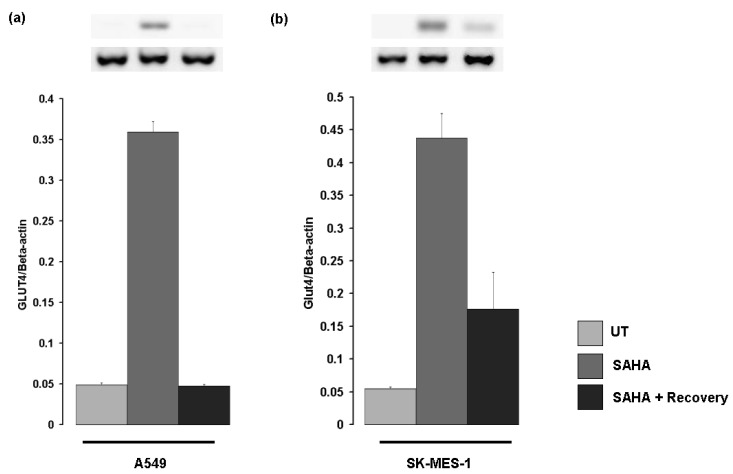
Detection of Glut-4 mRNA in cells following recovery from HDAC inhibition. Cells were treated for 24 hours with SAHA and either processed immediately or had fresh media without drug replaced and allowed to recover for a further 24 hours. (**a**) In A549 cells SAHA induced Glut-4 levels returned to basal levels following recovery. In contrast, (**b**) 24 hours post-recovery Glut-4 mRNA was readily detectable in SK-MES-1 cells (UT, untreated; SAHA, Vorinostat).

To determine if the induction of Glut-4 mRNA in response to histone deacetylase inhibition is sustained, cells were treated with SAHA for 24 hours and either processed directly or the media replaced and the cells allowed to recover for a further 24 hours. In A549 cells Glut-4 mRNA returned to basal levels within 24 hours (Figure 5a), while in SK-MES-1 a sustained elevation in Glut-4 mRNA is observed 24 hours following drug removal (Figure 5b).

### 2.6. Potential Regulation of Glut-4 by CTCF

**Figure 6.**
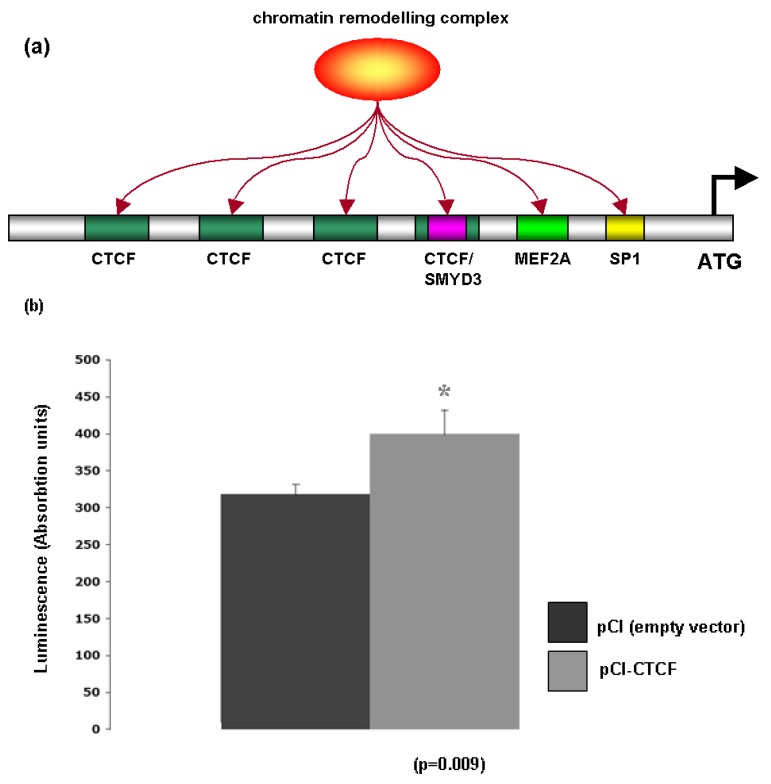
CTCF may be involved in the regulation of Glut-4 in NSCLC (**a**) Bioinformatic analysis of the Glut-4 promoter identified a series of binding sites for chromatin modifying proteins which include SMYD3, MEF2A, SP-1 and CTCF. (**b**) A plasmid construct containing the Glut-4 promoter luciferase construct (pHGT4−2031/+61), were transfected with either a CTCF overexpression construct (pCI-CTCF), or its corresponding empty vector (pCI), and luciferase activity measured to determine if CTCF may be involved with regulating Glut-4. Overexpression of CTCF was found to enhance luciferase activity in transfected cells compared to the empty vector control.

Bioinformatic analysis of the Glut-4 promoter revealed the presence of several consensus sites for proteins associated with chromatin remodeling including the lysine methyltransferase SMYD3A [OMIM: 608783], MEF2A [OMIM: 600660], Sp1 [OMIM: 189906] and CTCF [OMIM: 604167] (Figure 6a). To test whether Glut-4 may be regulated via CTCF, a luciferase construct fused to the Glut-4 promoter (pHGT4−2031/+61) was transfected into A549 cells in conjunction with either a CTCF overexpression vector (pCI-CTCF), or its corresponding empty vector (pCI), and luciferase activity measured 48 hours post transfection. The CTCF overexpression vector was found to enhance luciferase activity from the Glut-4 promoter (Figure 6b). CTCF is a highly conserved zinc finger protein that regulates gene transcription through the formation of higher order chromatin structures [23]. It has been postulated that CTCF may act as a transcription factor for context-dependent promoter activation/repression [23], and our results would seem to indicate that CTCF may be able to activate transcription of Glut-4. Support for this observation comes from a study of CTCF binding sites using high-resolution mapping of CTCF sites that identified a CTCF binding site within the Glut-4 promoter [24].

It is also well established that MEF2A regulates Glut-4 [25] in a process involving histone acetylation [26]. Indeed, in adipocytes it has been shown that MEF2A associates with histone deacetylase 5 to regulate Glut-4 expression [27]; further supporting our data that Glut-4 is regulated through dynamic chromatin remodeling.

### 2.7. Discussion

The insulin/IGF signaling pathways are key regulators of energy metabolism and growth and considerable evidence now exists that these hormones and the signal transduction networks they regulate have important roles in cancer [28,29]. Overexpression of the insulin receptor is associated with increased risk of metastasis and decreased survival in NSCLC [13], while the IGF1R is also emerging as an exciting candidate therapeutic target in this disease [30]. There is increasing evidence that regulation of the insulin signaling pathway is dynamically controlled using chromatin remodeling [31–33]. Glucose transporters, key regulators of cellular bioenergetics, are regulated by insulin/IGF signaling. Further evidence for this has come from studies linking histone acetylation and histone methylation with regulation of Glut-5 in colon cells [34].

The potential for using histone deacetylase inhibitors to target tumor bioenergetics through modulating glucose metabolism has recently been postulated for multiple myeloma, where cells treated with such inhibitors downregulated Glut-1 and inhibited hexokinase activity [35]. We were unable to detect any changes in Glut-1 expression in NSCLC cell lines, and as such directly targeting glucose metabolism through downregulating Glut-1 is not feasible in lung cancer. However, the data presented by us in this article clearly demonstrates that both Glut-3 and Glut-4 are dynamically regulated by histone post-translational modifications including histone acetylation. Conceivably, the ability of HDAC inhibitors to upregulate glucose transporters represents a potential mechanism for targeting solid tumors such as NSCLC through the Warburg effect. Metabolic targeting drugs based on glucose have been developed such as 2-deoxyglucose which show promise in targeting solid tumors [36]. Intriguingly this drug has been shown to enhance radiation- and chemotherapeutic drug-induced damage in a number of cancer cells under *in vitro* and *in vivo* conditions while sparing or protecting normal cells [37]. This raises the possibility that by inducing Glut isoforms in lung cancer patients and thus enhancing metabolic targeting, may also allow more aggressive radiation- and chemotherapeutic approaches to be considered.

We have demonstrated the induction of both Glut-3 and Glut-4 in NSCLC cell lines in response to HDAC inhibition. Glut-3 generally considered to be confined to tissues that exhibit a high glucose demand [38], while Glut-4 is an insulin dependent glucose transporter. This raises the exciting possibility of combining metabolic targeting drugs such as 2-deoxy-glucose (2-DG), epigenetic targeting therapies such as histone deacetylases and inhaled insulin to harness the potential of the Warburg effect in NSCLC. Our data showing that the induction of Glut-4 can be sustained after removal of HDACi raises the possibility of using staggered treatments to gain maximum benefit. Furthermore such therapies could conceivably also allow for more aggressive modalities to be used.

Inhaled insulin has been suggested as a potential means to detect early lung cancer [14]. Our data could potentially be utilized in this regard to enhance FDG-PET imaging for the early detection of lung cancer. Prior to imaging patients with suspected lung cancer could be treated with a histone deacetylase inhibitor to upregulate expression of GLUT transporters. One candidate HDACi which may be suitable is Phenylbutyrate (triButyrate®). This drug is currently FDA approved for the treatment of urea cycle disorders, and currently recommended dosage is in the order of 450 to 500 mg/kg bodyweight per day. Once in the body it is quickly metabolized to a naturally occurring metabolite of phenylalanine, one of the main reasons for its low toxicity.

On a more cautious note, both insulin and insulin-like growth factor 1 have recently been shown to have potent mitogenic effects on lung fibroblasts [39], and as such the development of therapies utilizing inhaled insulin should be essentially for short durations. It must be noted that this finding remains controversial as other studies have also shown that inhaled insulin does not trigger lung inflammation and airway remodeling [40].

## 3. Materials & Methods

### 3.1. Cell Lines and Primary Tumor Samples

NSCLC cell lines A549 (adenocarcinoma, ADC), and SK-MES-1 (squamous cell carcinoma, SCC) and H460 (large cell), were purchased from the ATCC (LGC Promochem). The Hep3B (Hepatocellular Carcinoma, HCC) cell line was a kind gift from Professor Pierre De Meyts (Hagedorn Research Institute, Copenhagen). HepG2 (HCC) and HEK-293 (human embryonic kidney) were a gift from Dr Matthew Lawless (UCD). H9C2 (muscle) were a kind gift from Dr Anthony Edwards (IMM, TCD). HK-2 cells were a kind gift from Dr Martin Leonard (Conway Institute, UCD). SGBS cells (human pre-adipocyte) [41] were a kind gift of Professor Martin Wabitsch.

All cell lines were maintained at a constant temperature of 37 °C in a humidified atmosphere of 5% CO2 in their appropriate cell culture media.

Twenty tumor specimens from patients presenting with Stages I and II resectable NSCLC at St. James's Hospital, Dublin were used. Matched normal tissue was taken in parallel for each patient and samples were evaluated by a pathologist immediately following dissection. Informed consent was obtained from each patient, and the study was conducted after formal approval from the Hospital ethics committee.

### 3.2. Cell Treatments with Trichostatin A, Phenylbutyrate, SAHA and 5-Aza-2′-deoxycytidine

Trichostatin A was purchased from Invivogen and dissolved in DMSO at a concentration of 250 mg/mL. Cell cultures were treated for 24 hours with Trichostatin A, at a final concentration of 250 ng/mL. Phenylbutyrate/Tributyrate™ (PB) was a generous gift from Triple Crown America. Cell cultures were treated with PB at a final concentration of 10 mM for 24 hours. SAHA (Zolinza/Vorinostat®) was purchased from Cayman Chemicals and dissolved in methanol. Cell cultures were treated for 24 hours at a final concentration of 5 μM. 5-Aza-2-deoxycytidine (Decitabine/Dacogen®) was purchased from Merck, and dissolved in ethanol. Cell cultures were treated for 48 h at a final concentration of 5 μM, with the media and drug replaced every 24 hours.

### 3.3. Nucleic Acid isolation

Total RNA was isolated using Tri-Reagent (MRC Gene) according to the manufacturer's instructions.

### 3.4. Generation of cDNA and Analysis of Gene Expression

1st strand cDNA was prepared from total RNA using Superscript II according to the manufacturer's instructions (Invitrogen). Expression of Gluts1-4 and Beta-Actin in A549 and SK-MES-1 cells was examined by RT-PCR using the primers and annealing conditions outlined using the following primers:

GLUT-1 For5′-CGGGCCAAGAGTGTGCTAAA-3′GLUT-1 Rev5′-TGACGATACCGGAGCCAATG-3′GLUT-2 For5′-CGTCTCCTTTCACATTTCCTTC-3′GLUT-2 Rev5′-GGTGGAGAAAACAGCCTAGAGAT-3′GLUT-3 For5′-CCAACTTCCTAGTCGGATTG-3′GLUT-3 Rev5′-AGGAGGCACGACTTAGACAT-3′GLUT-4 For5′-CCCCCTCAGCAGCGAGTGA-3′GLUT-4 Rev5′-GCACCGCCAGGACATTGTTG-3′GLUT-5 For5′-ATCCAGCCTGTTAGAAGAGACC-3′GLUT-5 Rev5′-TAGGGCGCGGCCGCGAAAAGTGATCAGGTTCAT-3′GLUT-11 For5′-CCTCAGCTTTGGCATTGGCCCTGCCGG-3′GLUT-11 Rev5′-GCCTTTGGTCTCAGGAAGGAACAGGCC-3′Beta-actin For5′-TGTTTGAGACCTTCAACACCC-3′Beta-actin Rev5′-AGCACTGTGTTGGCGTACAG-3′

PCR cycling conditions were as follows: 95 °C for 5 minutes followed by 30 cycles of (1 minute at 94 °C, 1 minute at 56 °C, 1 minute at 72 °C) with a final extension at 72 °C for 10 minutes. 10 μL of the Glut RT-PCR product and 2 μL of the Beta-Actin RT-PCR product were loaded onto a 1.2% agarose gel. Quantification of the RT-PCR results were obtained by scanning the gel images and importing the data into TINA 2.09c (Raytest, Isotopenmeβgeräte GmbH, Germany) with Beta-Actin levels utilized as the internal control in each case as appropriate. The values for the gene under scrutiny were then normalized to the internal control.

### 3.5. Chromatin Immunoprecipitation (X-ChIP)

Chromatin immunoprecipitation was performed as follows: Following treatments, cells were fixed with formaldehyde (final concentration 1%), suspended in SDS lysis buffer (Millipore) and sonicated until DNA was fragmented into lengths of between 200-1000. Aliquots of this sheared DNA were subsequently immunoprecipitated using the OneDay ChIP Kit™ (Diagenode) according to the manufacturer's instructions. The antibodies used for immunoprecipitations were as follows: pan acetyl-histone H3 (Millipore Cat#06-599), pan acetyl histone H4 (Millipore Cat# 06-598), histone H3K9/14ac (Diagenode Cat# pAb-ACHBHS-044), histone H3K9ac (Diagenode Cat# pAb-ACHAHS-044). histone H3K4Me3 (Diagenode Cat# pAb-003-050), histone H3K4Me2 (Sigma-Aldrich Cat# D5692), histone H3K4Me (Sigma-Aldrich Cat# M4819) and histone H3K9Me2 (Sigma-Aldrich Cat# D5567). 1 μg of antibody was used per immunoprecipitation as recommended by the manufacturers for the OneDay ChIP Kit™ (Diagenode). A no-antibody control was included to test for non-specific carriage of DNA with histones.

PCR primers for studying Glut-4 by ChIP were designed from the Glut-4 promoter sequence taken from TRED (ID: 113625) [42]. The primers used were as follows:

GLUT-4 ChIP Fwd5′-GTGTCTCGCCAGCCACGCGG-3′GLUT-4 ChIP Rev5′-GGGCTGGGGGAAAAGACGCG-3′

PCR conditions are the same as described in *Section 3.4*

### 3.6. CTCF Luciferase Assay

The plasmids pCI (empty vector) and pCI-CTCF were a gift from Dr. Elena Klenova (University of Essex, Essex, United Kingdom). The Glut-4 promoter luciferase construct (pHGT4-2031/+61) was generously provided by Dr Yong-Ho Ahn (Yonsei University College of Medicine, Seoul, Korea). Transient transfections of pCI and pHGT4−2031/+61, or pCI-CTCF and pHGT4−2031/+61 were performed using Fugene 6 reagent (Roche) according to the manufacturer's instructions. After 48 hours, cells were washed with PBS and lysed in 150 μL of reporter lysis buffer (Promega). Luciferase activities were measured using the Luciferase Assay System (Promega).

## 4. Conclusions

Our results have confirmed that Glucose transporters can be induced in NSCLC using epigenetic targeting. We have shown that both Glut-3 and Glut-4 can be induced in this fashion, and that such responses can be sustained following removal of drug. Our data suggests that this may be a unique way to metabolically target NSCLC which may have important clinical implications by allowing for more aggressive therapies to be considered. Furthermore, the ability to upregulate the insulin dependent glucose transporter Glut-4 may be of clinical benefit in the early detection of lung cancer as a combination of HDACi and inhaled insulin may enhance FDG-PET imaging.

## References

[1] Garber K. (2006). Energy deregulation: licensing tumors to grow. Science.

[2] Ganapathy V., Thangaraju M., Prasad P.D. (2009). Nutrient transporters in cancer: Relevance to Warburg hypothesis and beyond. Pharmacol. Ther..

[3] Busk M., Horsman M.R., Kristjansen P.E., van der Kogel A.J., Bussink J., Overgaard J. (2008). Aerobic glycolysis in cancers: Implications for the usability of oxygen-responsive genes and fluorodeoxyglucose-PET as markers of tissue hypoxia. Int. J. Cancer.

[4] Uldry M., Thorens B. (2004). The SLC2 family of facilitated hexose and polyol transporters. Pflugers Arch..

[5] Simpson I.A., Dwyer D., Malide D., Moley K.H., Travis A., Vannucci S.J. (2008). The facilitative glucose transporter GLUT-3: 20 years of distinction. Am. J. Physiol. Endocrinol Metab..

[6] Brown R.S., Leung J.Y., Kison P.V., Zasadny K.R., Flint A., Wahl R.L. (1999). Glucose transporters and FDG uptake in untreated primary human non-small cell lung cancer. J. Nucl. Med..

[7] Wood I.S., Trayhurn P. (2003). Glucose transporters (GLUT and SGLT): Expanded families of sugar transport proteins. Br. J. Nutr..

[8] Birnbaum M.J. (1989). Identification of a novel gene encoding an insulin-responsive glucose transporter protein. Cell.

[9] Garvey W.T., Maianu L., Zhu J.H., Brechtel-Hook G., Wallace P., Baron A.D. (1998). Evidence for defects in the trafficking and translocation of GLUT-4 glucose transporters in skeletal muscle as a cause of human insulin resistance. J. Clin. Invest..

[10] Devaskar S.U., deMello D.E. (1996). Cell-specific localization of glucose transporter proteins in mammalian lung. J. Clin. Endocrinol. Metab..

[11] Ito T., Noguchi Y., Udaka N., Kitamura H., Satoh S. (1999). Glucose transporter expression in developing fetal lungs and lung neoplasms. Histol. Histopathol..

[12] Ito T., Noguchi Y., Satoh S., Hayashi H., Inayama Y., Kitamura H. (1998). Expression of facilitative glucose transporter isoforms in lung carcinomas: its relation to histologic type, differentiation grade, and tumor stage. Mod. Pathol..

[13] Muller-Tidow C., Diederichs S., Bulk E., Pohle T., Steffen B., Schwable J., Plewka S., Thomas M., Metzger R., Schneider P.M., Brandts C.H., Berdel W.E., Serve H. (2005). Identification of metastasis-associated receptor tyrosine kinases in non-small cell lung cancer. Cancer Res..

[14] Lehrer S. (2008). Inhaled insulin for the early detection of lung cancer. Med. Hypotheses.

[15] Lawless M.W., Norris S., O'Byrne K.J., Gray S.G. (2009). Targeting histone deacetylases for the treatment of disease. J. Cell. Mol. Med..

[16] Raychaudhuri N., Raychaudhuri S., Thamotharan M., Devaskar S.U. (2008). Histone code modifications repress glucose transporter 4 expression in the intrauterine growth-restricted offspring. J. Biol. Chem..

[17] Gray S.G., Qian C.N., Furge K., Guo X., Teh B.T. (2004). Microarray profiling of the effects of histone deacetylase inhibitors on gene expression in cancer cell lines. Int. J. Oncol..

[18] Berger S.L. (2007). The complex language of chromatin regulation during transcription. Nature.

[19] Sims R.J., Millhouse S., Chen C.F., Lewis B.A., Erdjument-Bromage H., Tempst P., Manley J.L., Reinberg D. (2007). Recognition of trimethylated histone H3 lysine 4 facilitates the recruitment of transcription postinitiation factors and pre-mRNA splicing. Mol. Cell..

[20] Pinskaya M., Morillon A. (2009). Histone H3 lysine 4 di-methylation: A novel mark for transcriptional fidelity?. Epigenetics.

[21] Pekowska A., Benoukraf T., Ferrier P., Spicuglia S. (2010). A unique H3K4me2 profile marks tissue-specific gene regulation. Genome Res..

[22] Black J.C., Whetstine J.R. (2011). Chromatin landscape: Methylation beyond transcription. Epigenetics.

[23] Phillips J.E., Corces V.G. (2009). CTCF: master weaver of the genome. Cell.

[24] Barski A., Cuddapah S., Cui K., Roh T.Y., Schones D.E., Wang Z., Wei G., Chepelev I., Zhao K. (2007). High-resolution profiling of histone methylations in the human genome. Cell.

[25] Liu M.L., Olson A.L., Edgington N.P., Moye-Rowley W.S., Pessin J.E. (1994). Myocyte enhancer factor 2 (MEF2) binding site is essential for C2C12 myotube-specific expression of the rat GLUT-4/muscle-adipose facilitative glucose transporter gene. J. Biol. Chem..

[26] Mukwevho E., Kohn T.A., Lang D., Nyatia E., Smith J., Ojuka E.O. (2008). Caffeine induces hyperacetylation of histones at the MEF2 site on the Glut-4 promoter and increases MEF2A binding to the site via a CaMK-dependent mechanism. Am. J. Physiol. Endocrinol. Metab..

[27] Sparling D.P., Griesel B.A., Weems J., Olson A.L. (2008). GLUT-4 enhancer factor (GEF) interacts with MEF2A and HDAC5 to regulate the GLUT-4 promoter in adipocytes. J. Biol. Chem..

[28] Pollak M. (2008). Insulin and insulin-like growth factor signalling in neoplasia. Nat. Rev. Cancer.

[29] Gray S.G., Stenfeldt Mathiasen I., De Meyts P. (2003). The insulin-like growth factors and insulin-signalling systems: An appealing target for breast cancer therapy?. Horm. Metab. Res..

[30] Gridelli C., Rossi A., Bareschino M.A., Schettino C., Sacco P.C., Maione P. (2010). The potential role of insulin-like growth factor receptor inhibitors in the treatment of advanced non-small cell lung cancer. Expert Opin. Investig. Drugs.

[31] Lawless M.W., O'Byrne K.J., Gray S.G. (2009). Histone deacetylase inhibitors target diabetes via chromatin remodeling or as chemical chaperones?. Curr. Diabetes Rev..

[32] Lawless M.W., Norris S., O'Byrne K.J., Gray S.G. (2009). Targeting histone deacetylases for the treatment of immune, endocrine & metabolic disorders. Endocr. Metab. Immune Disord. Drug Targets.

[33] Gray S.G., De Meyts P. (2005). Role of histone and transcription factor acetylation in diabetes pathogenesis. Diabetes. Metab. Res. Rev..

[34] Inamochi Y., Mochizuki K., Osaki A., Ishii T., Nakayama T., Goda T. (2010). Histone H3 methylation at lysine 4 on the SLC2A5 gene in intestinal Caco-2 cells is involved in SLC2A5 expression. Biochem. Biophys. Res. Commun..

[35] Wardell S.E., Ilkayeva O.R., Wieman H.L., Frigo D.E., Rathmell J.C., Newgard C.B., McDonnell D.P. (2009). Glucose metabolism as a target of histone deacetylase inhibitors. Mol. Endocrinol..

[36] Dwarakanath B.S. (2009). Cytotoxicity, radiosensitization, and chemosensitization of tumor cells by 2-deoxy-D-glucose in vitro. J. Cancer Res. Ther..

[37] Farooque A., Afrin F., Adhikari J.S., Dwarakanath B.S. (2009). Protection of normal cells and tissues during radio- and chemosensitization of tumors by 2-deoxy-D-glucose. J. Cancer Res. Ther..

[38] Gould G.W., Holman G.D. (1993). The glucose transporter family: structure, function and tissue-specific expression. Biochem. J..

[39] Warnken M., Reitzenstein U., Sommer A., Fuhrmann M., Mayer P., Enzmann H., Juergens U.R., Racke K. (2010). Characterization of proliferative effects of insulin, insulin analogues and insulinlike growth factor-1 (IGF-1) in human lung fibroblasts. Naunyn. Schmiedebergs. Arch. Pharmacol..

[40] Papagianni M., Hatziefthimiou A., Chachami G., Gourgoulianis K., Molyvdas P.A., Paraskeva E. (2009). Inhaled insulin does not trigger lung inflammation and airway remodelling. Eur. Respir. J..

[41] Wabitsch M., Brenner R.E., Melzner I., Braun M., Moller P., Heinze E., Debatin K.M., Hauner H. (2001). Characterization of a human preadipocyte cell strain with high capacity for adipose differentiation. Int. J. Obes. Relat. Metab. Disord..

[42] Jiang C., Xuan Z., Zhao F., Zhang M.Q. (2007). TRED: A transcriptional regulatory element database, new entries and other development. Nucleic Acids Res..

